# Exploration of constructs of professionalism identified in the ABIM framework as perceived by the faculty fitting the Pakistani context

**DOI:** 10.12669/pjms.36.3.1573

**Published:** 2020

**Authors:** Humaira Fayyaz Khan, Raheela Yasmeen

**Affiliations:** 1 Prof. Dr. Humaira Fayyaz Khan, FCPS, MHPE. Department Physiology, Riphah University, Islamabad, Pakistan; 2 Prof. Dr. Raheela Yasmeen, DCPS HPE, MHPE. Department of Medical Education, Riphah University, Islamabad, Pakistan

**Keywords:** Accountability, Altruism, Duty and excellence, Empathy, Honor and integrity, Professionalism, Respect

## Abstract

**Objective:**

Domains of professionalism are well-described in the literature. Examining the elements of Professionalism in the local context have received less attention from education experts. The aim of the study was to explore the construct of professionalism as perceived by the faculty that fitted the Pakistani context identified in the ABIM framework of professionalism.

**Methods:**

This qualitative ethnographic research was conducted involving nine participants from Islamic International Medical College in Riphah University Islamabad. A four hours Focus Group Discussion was undertaken to explore the views of the faculty. The focus group session was audiotaped, transcribed and technique of triangulation was employed. Shortened meaningful unit (SMU) were identified from the transcribed data and analyzed to make codes for themes for the behaviors. Forty-six meaningful units were categorized and codes were identified. The themes were identified under the domains of the ABIM frameworks for the Pakistani context.

**Results:**

The participants listed 2-8 elements for each domain of the framework describing the professional conduct which lead to 140 shortened meaningful units. These were organized into 46 higher order codes.

**Conclusions:**

The study concludes that that ABIM framework can be used to build consensus regarding the domains of professionalism. No difference was found cross contextually regarding the domains of ABIM framework of professionalism.

## INTRODUCTION

Professionalism comprises of a set of values underlying the social bond between the society and the medical profession and is understood as a complex and multi-dimensional construct.^1^ The world-wide transformation of the medical curriculum towards an integrated and competency-based approach has resulted in professionalism emerging as an essential and a sustained theme.^2^

Expectancies from an undergraduate exiting medical student are many; such as respect for peers, teachers, lab staff, patients and their attendants, paramedical staff, at the level of undergraduate medical education.^3^ Questions regarding professionalism can arise because of lack of awareness regarding morals, professional code of conducts and law in health sciences. Because of the unpredictability of situations calling for wisdom and technical ability, there is worldwide agreement to inculcate professional principles in students of medicine from the start of their education bridging the gap between medical science and society.^4^ Failure of recognition of these values can severely hamper the patient-professional relationship and should be addressed in training program for graduating medical students.

The literature identifies different qualities to be seen in undergraduate medical students for exhibition of professionalism. According to the American board of Internal Medicine (ABIM) framework which has been mainly developed for the western context there are broadly six domains for the students to show their behaviors and attribute of professionalism. The central values are Honesty and integrity, Altruism, Respect, accountability, empathy, duty and excellence.^3^ The six domains of Professionalism as described by the American board of Internal Medicine (ABIM) was the focus of present study. There can be many similarities between professional behavioral and attributes still they can be context specific due to cross-cultural differences.^5^ In a Muslim society which has its basis on faith in Allah almighty these can be seen as internal motivators.^6^

Professionalism is being taught vertically in Islamic International Medical College during the five years of medical education. Literature concerning the elements of medical students in the local context is limited.^7^ The necessity is to address this topic separately as the final year student have not entered into active profession but have learnt professionalism during their undergraduate years. For this student need to be assessed for professionalism as a competency as they exit the final year of medical studies. This requires an agreement amongst the faculty about aspects of professionalism that needs to be exhibited by the students.

Examining the elements of Professionalism in the local context have received less attention from education experts. Thus, the aim of the study was to explore the construct of professionalism as perceived by the faculty that fitted the Pakistani context identified in the ABIM framework of professionalism.

## METHODS

This qualitative ethnographic research was conducted in Riphah University, Islamabad after the approval of Ethical Review Committee (Ref # Riphah/ERC/18/0260, dated February 6, 2018). Relevant literature search was done by the strategy recommended by Haig and Dozier.^8^ The electronic data base search was conducted using search engines such as PubMed, ERIC, Google scholar to determine if measures of the construct for measuring professionalism already existed in the local context. The method employed for inquiry into literature search was through key words and phrase with Boolean commands “AND” and “OR”. The inclusion criteria were original articles, review articles, articles in English and all articles relevant to search. Whereas, studies that were not in English language, studies with citations only, letters to the editor and those that were not relevant to the project were excluded.

Six open-ended questions for the focus group were developed around the domains identified in ABIM framework in the literature for data collection. The questions were sent to medical educationists of the college for validation. Changes suggested by the medical educationist were incorporated in the questions and were finalized.

### Focus group Discussion

All clinical faculty members who fulfilled the inclusion criteria of being certified medical educationist and having at least ten years of experience of supervising the final year undergraduate medical students were invited for the discussion. They were sent an invitation for the focus group via email explaining the rationale of the study and the question for focus group. A total of nine faculty members reported for the focus group. The least number of participants for a focus group discussion is six, therefore the discussion was carried on.^9^ A research team comprising of two faculty members with medical education background was constituted to help in the data collection and the process of focus group which was audio recorded.

Focus group discussion was conducted after informed consent. A PowerPoint with a brief overview of the project and the questions was shown to the participants. The format of the questions was open-ended so as to motivate participants to elaborate on the topic to increase the richness of the data.

The duration of focus group discussion was four hours. The age group of the participants was between 45-60 years and were from the specialties of Surgery, Medicine, Obs and Gyn, Paeds, Eye and ENT. Technique of triangulation was employed to bring credibility to the results.

### Data analysis

The recorded data was transcribed on to word document. Data was analyzed manually. The qualities described were color coded. Notes taken by the two members of the research team. Data was condensed and merged into short meaningful unit (SMU). These shortened meaningful units were analyzed to make codes for themes for the behaviors. The codes were then linked to the domains of professionalism.

## RESULTS

The demographic variable are given in Table-I. In response to the open-ended questions the participants listed 2-8 element of each domain describing the professional conduct desired to be seen in the final year medical students in the local context. These were sorted into 140 shortened meaningful units. They were further categorized into 46 higher order codes which were organized around the themes of the domains of the ABIM framework. The codes were classified to portray the desired characteristics which were deemed necessary by the clinician to be exhibited by a final year student. The participants did not add any new element to the ABIM domains. Representative statements of the behaviors and attitudes for the students to be exhibited by students are given in Table-II. The most important codes identified were from the domain of respect.

**Table-I:** Demographic variables.

Variable	
***Gender***	
Male	4
Female	5
***Year’s as clinical supervisor***	
Participant 1	Senior
Participant 2	Junior
Participant 3	Senior
Participant 4	Senior
Participant 5	Junior
Participant 6	Junior
Participant 7	Senior
Participant 8	Senior
Participant 9	Senior
Age (mean)	53 ± 6 years
***Specialties***	
Medicine	2
Surgery	2
ENT	1
Obstetrics and Gynae	1
Psychiatry	1
Paediatrics	1
Academic	1

Seniority is attributed to a professional with experience of more than 15 years in supervision and teaching of undergraduate medical students.

## DISCUSSION

This study was undertaken to explore the views of faculty regarding the professionalism of the exiting clerkship students as identified around the ABIM framework in Pakistani context. There was an agreement amongst all the participants that ABIM framework can be used to build consensus regarding the domains of professionalism. This is in contrast to a Persian study in which only three elements of the framework of professionalism were found to be valid for the Iranian context.^10^ However, that was a pilot study through a 17-item questionnaire.

The element of professionalism which was the most important in the local context was respect. This domain was the most emphasized as it represented an overall approach of the students towards patients, peers, faculty and health care staff workers. The cultural aspects may make the patient feel that he is not being respected or being treated professionally during interaction with the medical students. It was agreed by the participants that in context of Asian and especially in Pakistani culture with different ethnicities Islam is a common religion which can guide and influence the behavior of doctors. This is in accordance with a study from the Arabian context.^11^ The participants agreed that student can show respect by completing assignment on time arriving, on time being respectful to the patients and their relatives, peers’ teachers and faculty. It can be displayed during student-patient interaction, if the student asks permission regarding examination of the patient. In other words, this element of professionalism is the reason students should manifest the desired attributes and behaviors as it emphasizes the importance of interpersonal and ethical competencies as being knowledgeable and skillful is insufficient for the medical profession. It is noteworthy that all the participants made a point that the students should be respecting the diversity of the society and show religious tolerance. This aspect of the focus group was in accordance with the views of a study by Abdel-Razig S et al. However, in that qualitative study internet technique was employed to gather data and was verified through the Delphi method.^5^

**Table-II:** Representative statements of the behaviors and attitudes by the participants.

Sr. No	No of ‘Short meaningful units’	Representative ‘Short meaningful units’ from participants quotes (SMU)	Codes identified from SMU	Number of codes identified	Sub-themes	Domains or Major Themes
**1.**	146	R1: “should honor their integrity and not take patients as a teaching material”	Int	17	Patients	
1		R2: “should take care of his religious and cultural background, of the patient”.	C			
1		R1: “respect the patient convenience while interacting”	Con			
1		R1: “treat the patient as a person”	Ca			
1		R2: “encourage the patient to talk and not to snub them”.	A l			
1		R3: “Student should be sensitive”	Sen			
1		R1: “Respect his/her fellow students and should	Tol		Peers	
1		R3: “have a good working relationship”	Co			
1		R 8: “Supportive to the colleagues”.	Sup			
1		R3: “Should be truthful about an incidence of conflict”.	T			
1		R 5: “attentive during ward rounds”	At		Faculty	
1		R 3: “Should always be punctual”	P			
1		R4: Complete tasks with honesty	H			
1		R5: “deal politely with them and give tasks in a good manner”	Pol		Health care staff	
1		R3: “Should be friendly with the”.	Fr			
1		R6: “The students should give due respect what they deserve”.	R			
1		R9: “Should be non-judgmental and communicate with the relatives respectfully”.	Rd		Society	
2.		R7: “Should express pain on the pain of the patient”.	Em	6	Altruism	
1		R6: “make him comfortable”	Com			
1		R3: “The student has to be good listener.”	GL			
1		R7: “Should approach the patients empathetically”	In			
1		R4: “Stays late to deal with an emergency”	Ov			
1		R9: “Extends help to patients when needed.”	Hp			
3.		R1: “Student should exhibit punctuality”.	Pun	5	Honor and Integrity	
1		R 5: “Should complete tasks honestly”.	Ho			
1		R6: “Should not cheat”.	Ch			
1		R3: “Show tolerance when conflicts arise”.	Tl			
1		•R2: “be honest and truthful .. and should not cheat”.	Tr			
4.		•R1: “Should act as a bridge between patients and clinician”.	Br	6	Duty	
1		•R3: “Completed the assignments and brought original material”.	Res			
1		•R5: “For best patient outcome should exhibit teamwork”.	Tw			
1		•R4: “Works collaboratively with other student”.	Cb			
1		•R8: “Complete assignments”.	Ass			
1		•R7: “Comes prepared for ward rounds”.	Pp			
5.		R1: “if a mistake is committed the student should ask forgiveness.”	For	4	Accountability	
1		R7: “Enthusiastic about his studies and asking for guidance”.	En			
1		R6: “Reflect upon their activities”.	Ref			
1		R4: “performances need to be recorded and accounted for in the assessment”	Acc			
6.		RE 2: “Appropriately dressed as they are representing a respectable profession”.	Dr	8	Excellence	
		R7: “Participate in wards patient care”.	Par			
		R4: “Time spent by the student in the ward”.	Pre			
		R8: “Is able to accept feedback by the faculty”.	Fe			
		R2: “On time for ward round”.	OT			
		R2: “Corrects his mistakes”.	Cr			
		R8: “Should be able to show interest in the patient welfare”.	It			
		R 1: “Should do hard work for excellence”.	Ex			
7.		Total categories	46		

Altruism which is selflessness and encompasses empathy can be shown by the students through an impartial attitude to the patient if he has some problem.^12^ This is helped by being considerate and respecting the culture and wishes of the patient. In the context of the local culture it includes the close relative engaging especially the elders of the patient in decision making if a diagnostic and therapeutic procedure has to be carried out. According to the participants this can be shown to the patient by not imposing their own wishes on the patients and through humanitarian activities. This is in accordance with the views of participants of a study, conducted by Kusumati et al. in Indonesia, outlining this aspect of professionalism in the same terms.^13^

During the FGD the element of accountability overlapped with duty to be performed by the students. In a Muslim society like Pakistan, the ultimate authority to be accountable is Allah the Almighty. If the students have that realization in their hearts, they will be inherently accountable for their actions and will perform their duties in good faith unquestioningly. It is cultural norm to be late in the local context, students think it is acceptable to be late for 10-15 minutes for the classes or for the ward rounds. This effects their punctuality and accountability. If they are on time the students will be able to self-regulate themselves and their responsibilities. This aspect of FGD was in accordance with a study by Ho and Al-Eraky which was done in the Arabian context which states that if the medical students become self-accountable it can lead to tangible improvement in professional conduct.^14^ The perceptions of the faculty were explored on the construct of professionalism in the local context around the domains of ABIM framework. Professionalism is intimately connected to the belief system of context, which in case of Pakistan is Islam. It resides in the very core of individuals acting as a moral compass.^11^ The behavior in context of professionalism refers to observable and measurable behavior reflecting professional standards and values. The inclusion of its concept in evaluation can apprise the faculty about actions of the students may they be right or wrong, ensuring that students are equipped with the responsibility that accompanies professional status.

Medical professionalism aims at preparing the exiting students to be able to work in complex hospital environment. Excellence entails a conscientious effort to exceed ordinary expectations and to make a commitment to life-long learning. After respect, this element was the most discussed and ranked highly by the group participants. A key in this is the pursuit of commitment to provide the highest quality of health care through life-long learning education and an individual who can reflect and is able to accept feedback. It was endorsed by all the participants as an essential quality to be exhibited by the student. This was in accordance to a study by Al-Eraky who proposed the four gates model and attributed excellence as the third gate in which becoming a professional doctor is not a destination but a life long journey.^15^

The perceptions of the participants matched with the competencies which have been identified in the revised PMDC curriculum which is being implemented in the Pakistani medical colleges. The framework of PMDC curriculum commits that a medical graduate should be aware of the competencies and fitness to practice guidelines and these characteristic should be exhibited so that the public trust can develop in them.^16^

### Limitations of the study

Results should be considered in the local setting and has certain limitations which are pertinent to qualitative research. Firstly, purposive sampling was employed which did not include the student representative, so the focus group discussion could be biased towards view point of the faculty. Secondly, due to inclusion of the minimum number of participants it is possible saturation point could not have been reached. Future studies could include the student perspective by including the student for their point of views.

## CONCLUSION

The study is likely to contribute to knowledge of medical educators in construction of framework of professionalism as they seek to understand, teach and assess it in local context. The participants rated all the attributes for professionalism which are Honesty, integrity, Altruism, Respect, Responsibility, accountability, Duty and excellence as important and no difference was found across culture regarding the basic elements.

### Implications

To the best of our knowledge this is first of its kind in which an effort is being made to validate a conceptual framework of professionalism for local context. The themes can be used as a baseline for developing an assessment tool for exiting student in the local context therefore it is critical to know the perceptions of the assessing faculty.

### Authors’ Contribution:

**HFK** conceived, designed, data collection and did statistical analysis.

**RY** statistical analysis and editing of manuscript.
